# Nasal Colonizers from Sows in the Federal District of Brazil Showed a Diverse Phenotypic Resistance Profile

**DOI:** 10.3390/microorganisms13061354

**Published:** 2025-06-11

**Authors:** Luciana Lana Rigueira, Fabiano José Ferreira de Sant’Ana, Bruno Stéfano Lima Dallago, Rômulo Salignac Araújo de Faria, Maurício Macedo Rodrigues, Pau Obregon-Gutierrez, Virginia Aragon, Simone Perecmanis

**Affiliations:** 1 School of Agricultural and Veterinary Sciences, Brasília University (UnB), Brasilia 70910-000, Brazil; santanafjf@unb.br (F.J.F.d.S.); dallago@unb.br (B.S.L.D.); faria.romulo@aluno.unb.br (R.S.A.d.F.); mauriciomacedo@unb.br (M.M.R.); perecmaniss@unb.br (S.P.); 2 School of Veterinary Sciences, Catholic University of Brasília, Brasilia 71966-700, Brazil; 3 Unitat Mixta d’Investigació IRTA-UAB en Sanitat Animal, IRTA Programa de Sanitat Animal, Centre de Recerca en Sanitat Animal (CReSA), Campus de la Universitat Autònoma de Barcelona (UAB), 08193 Bellaterra, Spain; pau.obregon@irta.cat

**Keywords:** antimicrobial resistance, nasal microbiota, swine, metaphylaxis

## Abstract

Antimicrobial resistance (AMR) is a major public health concern influenced by antimicrobial use (AMU) in animal production systems. In swine, metaphylactic treatments may contribute to the emergence and dissemination of resistance genes. In this study, we isolated bacteria from the nasal cavities of 50 sows across 10 farms in the Federal District, Brazil. A total of 132 bacterial isolates were obtained and tested for phenotypic resistance to 23 antimicrobials using the disk diffusion method. Resistance was detected against all tested antimicrobials, with an overall resistance rate of 55.6% (1605/2888 tests). The highest resistance rates were observed for bacitracin (92.4%) and penicillin (79.2%), while lower resistance rates were found for aminoglycosides. Most isolates exhibited multidrug resistance to 7–9 classes of antimicrobials, including strains of *Staphylococcus*, *Escherichia coli*, and *Klebsiella*—all of which are relevant in the context of One Health. *Actinobacillus suis* showed the highest resistance levels among all identified species. AMR was positively correlated with both the duration and the number of antimicrobial agents used in feed, reinforcing the need for prudent AMU practices. The use of autogenous vaccines against *Pasteurella multocida* was associated with reduced lung lesions, underscoring the value of vaccination in disease control. AMR surveillance programs may benefit from including bacterial colonizers from the microbiota, though further studies are necessary to better understand the resistance dynamics of these commensals.

## 1. Introduction

Antimicrobial agents (AMBs) have long been used in livestock to prevent disease and promote growth, particularly in intensive swine and poultry production [1]. In Brazil, AMBs remain part of herd management strategies, especially during critical periods such as the dry season, to reduce disease incidence and maintain productivity [2]. However, widespread and prolonged antimicrobial use (AMU) has contributed to the selection and dissemination of antimicrobial-resistant (AMR) bacteria, posing a major challenge to both animal and public health [3,4].

Therefore, international organizations such as World Health Organization (WHO), Food and Agriculture Organization of the United Nations (FAO) and World Organisation for Animal Health (WOAH) have promoted the One Health approach, urging countries to strengthen biosecurity, animal welfare, and AMU surveillance [5]. Brazil has taken significant regulatory steps, including restrictions on growth-promoting AMBs and the implementation of the National Action Plan for the Prevention and Control of Antimicrobial Resistance in Agriculture in Brazil (PAN-BR-AGRO) program for monitoring AMU and AMR in animal production systems [6]. While progress has been made, challenges remain in aligning on-farm practices with these standards [7].

Efforts to reduce AMU include improving vaccination protocols, enhancing herd management, and promoting microbial balance to support host immunity [8]. Biosecurity plays a key role in these strategies, as stress and poor welfare and housing conditions can predispose animals to disease, increasing reliance on antimicrobials [9]. Monitoring AMR in clinically ill animals is essential to understand resistance patterns [10]. For respiratory diseases, the nasal microbiota serves as an interface for pathogen interaction and is critical for respiratory health [11]. However, colonization by resistant bacteria, even in the absence of disease, represents a hidden risk of AMR transmission within herds and to humans and the environment [12]. Data from respiratory clinical cases in Brazil showed that the bacteria with the highest levels of resistance were *Streptococcus suis* and *Bordetella bronchiseptica*. Additionally, 50% of *Glaesserella parasuis* isolates exhibited high resistance to the combination of sulfadiazine/trimethoprim [13]. These three bacteria can be found in the respiratory microbiota of pigs. Thus, monitoring the nasal commensal communities may represent a non-invasive procedure for surveillance of resistance in the production system.

Here, we report the isolation of some bacterial colonizers from the nasal microbiota of sows from farms with different sanitary management practices in the Federal District, Brazil and describe the phenotypic resistance profile of the isolates. Our findings contribute to a growing body of knowledge essential for guiding AMU strategies, reinforcing responsible antimicrobial practices in animal husbandry [14].

## 2. Materials and Methods

### 2.1. Ethical Approval

All samples were collected according to Animal welfare management—General requirements and guidance for organizations in the food supply chain—ISO/TS 34700:2016 [15]—with the permission of the farm owners. The study was approved by the Ethical Committee for the Use of Animals (CEUA) of the University of Brasília nº 23106.022976/2023-55.

### 2.2. Sampling, Swine Farms and Sow Health Management

Between March 2022 and October 2023, sows were sampled from 10 swine farms (A to J) in the Federal District, Midwest Brazil. Out of 24 swine farms in the region, 10 are sow farms categorized as two-site herd (1 farm), farrow-to-finish (3 farms), and one-site herd (6 farms). These farms varied in herd size (50 to 4273 sows) and health management practices. The same researcher interviewed and collected data in all participating herds.

The management practices vary between breeding stock replacement, quarantine protocols, vector control, feed type, sanitary barriers regarding human and vehicle entrance and sanitary practices. The sanitary practices vary between hygiene routines (use or omission of neutral soap before disinfection), sanitary downtime (3–5 days), animal welfare status (only Farm C was Animal Welfare Certified), biosecurity measures (see Table 1). Most farms use antimicrobials for metaphylatic treatments wich were also used for susceptibility testing (Table 2). Although there are no mandatory vaccines, the farms used commercial and autogenous vaccines with different protocols which differ among the farms (see Table 3). Regarding metaphylactic antibiotic use, all farms used in-feed amoxicillin for 4 months-cycle/year, while some farms also used other AMBs on a rotational basis in the sow feed (see Table 3). The farms used medicated feed to acclimate the sows as a preventive strategy to prevent atrophic rhinitis in sows and their offspring and thus control the spread of infectious diseases by reducing shedding. However, some farms added florfenicol, penicillin or tylosin, alternating the AMBs with each use, as recommended by the veterinarian. Farm managers used 800 mg/ton of amoxicillin to achieve 20 mg/kg per lactating sow. Amoxicillin is a broad-spectrum penicillin AMB that is active against many Gram-positive and some Gram-negative bacteria, including *Pasteurella multocida*, a major pathogen in atrophic rhinitis and *B. bronchiseptica* [16]. Farm A frequently included clindamycin, tetracycline, enrofloxacin, and oxytetracycline in the feed rotation for metaphylactic purposes, and Farm J included clindamycin in an attempt to increase the effectiveness of treatment. Occasionally, marbofloxacin, gentamicin, or amoxicillin with clavulanic acid was injected for therapeutic treatment. The use of amikacin was not reported by any of the farm managers or staff.

Five sows per farm, aged 14–48 months, were selected by convenience sampling. Sow batches ranged from 20 to 60 animals across farms. The cross-sectional study included 463 sows housed in the sampled batches. The total population of sows in the Federal District farms was 9544. Preference was given to sows with respiratory signs (coughing, prostration); if unavailable, apparently healthy sows from the same batch were sampled to reach the target number.

In total, 50 nasal swabs were collected in duplicate—one set was placed in sterile Falcon tubes for PCR, and the other in 5 mL Brain Heart Infusion—BHI (KASVI^®^) for bacterial culture. Slaughterhouse surveillance [17], conducted independently from the study, reported lung lesions and their etiology in pigs from the same farms.

### 2.3. Biosecurity Data Collection

Biosecurity was assessed using a structured questionnaire based on the official form from Brazil’s Ministry of Agriculture, Livestock, and Supply (MAPA). Ten key indicators were evaluated to score the risk of disease introduction and spread, including farm isolation, proximity to other herds and roads, breeding stock replacement, quarantine protocols, vector control, feed type, transportation practices, and access logging for vehicles and personnel. Each farm received a Biosecurity Score (Bio) ranging from 6 to 9 (median: 7.1) on a scale of 1 to 10, with higher scores indicating better preventive measures (see Table 1).

### 2.4. Culture and Bacterial Isolation

Bacterial isolation was performed at the Veterinary Microbiology Laboratory, University of Brasília (UnB), using standard microbiological methods. Nasal swabs were cultured on blood agar and incubated at 37 °C overnight. Species-level identification was performed using biochemical profiling following our laboratory’s Standard Operating Procedures. Colonies were characterized by morphology, hemolysis, Gram staining, catalase and oxidase activity. Gram-positive bacilli with spores and yeasts were excluded. Further identification included oxidative/fermentative (OF), methyl red, and Voges–Proskauer (Vm/Vp) tests. Additional tests followed laboratory Standard Operating Procedures. *Staphylococcus* spp. were cultured on mannitol salt agar and identified using the coagulase test. Enterobacteria were characterized using indole, citrate, urea, and triple sugar iron (TSI) tests, as well as assessments of sugar and protein metabolism. Confirmed isolates were plated on blood agar and stored in Brain Heart Infusion (BHI) with 20% glycerol at −80 °C.

### 2.5. Antimicrobial Susceptibility Testing

Phenotypic antimicrobial resistance (AMR) was evaluated using the Kirby–Bauer disk diffusion method [18]. Bacterial isolates were classified as susceptible (S), intermediate (I), or resistant (R) based on the diameter of inhibition zones, measured with a pachymeter. Interpretations followed Clinical and Laboratory Standards Institute (CLSI) guidelines [19]. Intermediate (I) results were considered susceptible, since they indicate potential effectiveness with increased antimicrobial exposure. A total of 23 antimicrobials were tested (see Table 2).

### 2.6. DNA Extraction and PCR Assays

Genomic DNA was extracted from nasal swab samples using the Genomic DNA Extraction Kit^®^ (Biogene, Madison, WI, USA), following the manufacturer’s protocol. PCR assays were performed as previously described [17]. Multiplex PCR was used to detect *Actinobacillus pleuropneumoniae*, *G. parasuis*, and *P. multocida*, using primers: AP-IVF ATACGGTTAATGGCGGTAATGG and AP-IVR ACCTGAGTGCTCACCAACG (346 bp) for *A. pleuropneumoniae* (apxIVA); KMT1 T7 ATCCGCTATTTACCCAGTGG and KMT1 SP6 GCTGTAAACGAACTCGCCAC-3′ (460 bp) for *P. multocida* (kmt1); and HPS-F GTGATGAGGAAGGGTGGTGT and HPS-R GGCTTCGTCACCCTCTGT (821 bp) for *G. parasuis* (16S rRNA) detection. The reaction mix (50 µL) consisted of 10 µL DNA, 1.25 U Taq polymerase, 1× buffer, 2 mM MgCl_2_, 200 µM dNTPs, 30 pmol primers. Cycling conditions were 5 min at 95 °C, 29 cycles of 30 s at 94 °C, 30 s at 58 °C and 45 s at 72 °C, with a final extension of 7 min at 72 °C. *Mycoplasma hyopneumoniae* was detected via nested PCR [19], which consisted of a first amplification with primers: A-F 5′-GAG CCT TCA AGC TTC ACC AAG A-3′/B-R 5′-GTG TTA GTG ACT TTT GCC ACC-3′ (649 bp) and a second amplification with primers: C-F 5′-ACT AGA TAG GAA ATG CTC TAG T-3′/D-R 5′-GTG GAC TAC CAG GGT ATC T-3′ (352 bp). The reaction mix (50 µL) consisted of 5 µL DNA, 1 U Taq, 1× buffer, 0.75 mM MgCl_2_, 200 µM dNTPs, 10 pmol primers. Cycling conditions were 3 min at 95 °C, 35 cycles of 1 min at 95 °C, 1 min at 64 °C for the first amplification or 60 °C for the second amplification, and 90 s at 72 °C, with a final extension of 5 min at 72 °C. Positive control samples, consisting of known strains of *A. pleuropneumoniae* (ATCC 27090), *G. parasuis* (ATCC 19417), *P. multocida* (ATCC 12945), and *M. hyopneumoniae* (ATCC 25934), were kindly provided by the Federal University of Viçosa (UFV) and used to validate the respective PCR assays. PCR products were visualized under UV light after electrophoresis in 1% agarose gels stained with GelRed™.

### 2.7. Statistical Analyses

Data visualization was performed in RStudio (version 2024.04.0) [20] using the ggplot2 package (version 3.4.0) [21]. Statistical analyses were conducted using SAS software (version 9.4, Cary, NC, USA). For each, antimicrobial resistance (AMR) was calculated as the proportion of resistant isolates among those tested. Associations between AMR and health management variables (e.g., vaccination protocols) were evaluated using the Chi-square test. Fisher’s exact test was applied when expected frequencies were below five. Pearson’s correlation was used to assess relationships between years of AMU the number of AMBs used, and the biosecurity score in relation to overall resistance per farm. Differences in resistance levels among bacterial species or across farms were analyzed using the Kruskal–Wallis test followed by Dunn’s post hoc test.

## 3. Results

### 3.1. Sampling Sow Data

Samples were collected from sows of different parity, with 42% (21/50) in first parity, 36% (18/50) between second and fourth parity and 22% (11/50) in fifth parity or above. A total of 54% (27/50) of the sows were in maternity stalls and 46% were in gestation stalls. A total of 44% (22/50) of the sows showed no clinical signs of infectious disease, but a history of infectious disease was reported in 34% (17/50) of the sows. During the visits, 22% (11/50) of the sows were coughing, sneezing and/or had purulent or sanguinolent nasal secretions.

The vaccination protocols varied among the farms. Commercial vaccines were used in 7/10 farms and were directed against *M. hyopneumoniae* (*Myo*), porcine circovirus type II (PCV2), *P. multocida*, *B. bronchiseptica*, *Salmonella* ser. Typhimurium, *S. suis* infection diseases. Autogenous vaccines were used in 8/10 herds to prevent outbreaks caused by *P. multocida*, *G. parasuis*, *S*. Typhimurium, *E. coli* and, *S. suis*. Table 3 details health management practices among swine farms, including metaphylactic feed and vaccine protocols adopted by each farm.

### 3.2. Lung Lesion Slaughter House Report and Pathogen Detection by PCR or Bacterial Culture in Nasal Samples from Sows

The abattoir report was produced independently of the study as part of a routine screening in official cooperation between industry and the veterinary service. All the isolates were obtained from the nasal samples obtained from the farms. This complementary data was useful to infer possible associations based on a combination of diagnostic findings (PCR and/or culture) and slaughterhouse reports as well as farm-level data on vaccine use and lesion prevalence. Associations between pathogen detection and lung lesions were inferred from the integration of diagnostic, epidemiological, and slaughter data, rather than from direct lesion-specific microbiological or histopathological confirmation.

Lung lesions caused by *P. multocida* were reported by the slaughterhouse in the herds of farms B, D and G, which did not use the corresponding vaccine. In nasal samples, *P*. *multocida* was detected by PCR and culture in farms A, B, E, G, H, and PCR but not culture in farm D.

As shown in Table 3, six of the ten farms applied *P. multocida* vaccines; five used autogenous vaccines and one a commercial one (farm C). Only one of them, farm J, reported lung lesions at slaughter (although isolation showed *A. pleuropneumoniae* in the lesions). The use of an autogenous vaccine against *P. multocida* acted as a protective factor against lung lesions, as this vaccine was significantly associated with a reduction in pulmonary lesions (*p*-value of 0.048). *P. multocida* was detected in 6 out of 10 farms and *G. parasuis* in 4 out of 10 farms. *G. parasuis* and *P. multocida* were detected together in one sow. *A. pleuropneumoniae* was detected only in farm J. *M. hyopneumoniae* was not detected in any of the 50 samples. Farm C had *Mhyo-free* status, but we cannot exclude the presence of *M. hyopneumoniae* in the other farms that tested negative by nested PCR. *G. parasuis* and *A. pleuropneumoniae* were only detected by PCR, as they cannot grow on blood agar plates. *A. pleuropneumoniae* was also detected in the slaughterhouse in samples from farm J. Detection of pathogens is summarized in Table 4.

### 3.3. Resistance Profile of Bacterial Isolates from the Sows’ Nasal Microbiota

A total of 132 bacterial strains, representing 20 species, were isolated from nasal swabs collected from 50 sows. The most prevalent isolates were coagulase-negative *Staphylococcus* spp., commensals commonly found in the nasal microbiota (22/50; 44%). To investigate AMB resistance in the isolates, tests with 23 AMB agents were performed (Supplementary Table S1). The overall AMR rate was 55.6% (1605/2888 tests), with notable differences among antimicrobial classes (Supplementary Tables S1 and S2). The isolates exhibited a low resistance rate to aminoglycosides (16.7–39.4%; Table S2), whereas extremely high resistance was observed for the polypeptide bacitracin (92.4%; Tables S1 and S2). Among the macrolides, tulathromycin showed the lowest resistance rate (33.6%), while erythromycin and tylosin exhibited higher rates (57.4% and 72.3%, respectively). Resistance to penicillin, a beta-lactam, was also high (79.2%). Notably, the resistance rate to amoxicillin was reduced from 65.2% to 27.3% when combined with the β-lactamase inhibitor clavulanic acid (Tables S1 and S2). It is also of note to highlight the presence of resistance to ceftiofur (a third-generation cephalosporin) in several taxa, including *A. suis*, *S. aureus* and *S. suis*.

A total of 132 bacterial strains, representing 20 species, were isolated from nasal swabs collected from 50 sows. The most prevalent isolates were coagulase-negative *Staphylococcus* spp., commensals commonly found in the nasal microbiota (22/50; 44%). To investigate AMB resistance in the isolates, tests with 23 AMB agents were performed (Supplementary Table S1). The overall AMR rate was 55.6% (1605/2888 tests), with notable differences among antimicrobial classes (Supplementary Tables S1 and S2). The isolates exhibited a low resistance rate to aminoglycosides (16.7–39.4%; Table S2), whereas extremely high resistance was observed for the polypeptide bacitracin (92.4%; Table S1 and Table S2). Among the macrolides, tulathromycin showed the lowest resistance rate (33.6%), while erythromycin and tylosin exhibited higher rates (57.4% and 72.3%, respectively). Resistance to penicillin, a beta-lactam, was also high (79.2%). Notably, the resistance rate to amoxicillin was reduced from 65.2% to 27.3% when combined with the β-lactamase inhibitor clavulanic acid (Tables S1 and S2). It is also of note to highlight the presence of resistance to ceftiofur (a third-generation cephalosporin) in several taxa, including *A. suis*, *S. aureus* and *S. suis*.

Although members of the *Enterobacteriaceae* family are typically associated with the intestinal microbiota, several genera—including *Klebsiella*, *Pantoea*, *Proteus*, *Yersinia*, *Escherichia*, and *Salmonella*—were also identified in the nasal samples. This is notable, as *Enterobacteriaceae* are named for their intestinal niche (from Greek enteron, meaning intestine) [22,23]. Among these, *Klebsiella* and *Escherichia coli* are relevant for their pathogenic potential and their recognized roles in antimicrobial resistance transmission within the One Health framework. In our study, both species exhibited multidrug resistance profiles, with resistance to β-lactams, tetracyclines, and sulfonamides, reinforcing their potential as reservoirs of resistance genes at the human–animal–environment interface.

Resistance levels correlated with AMU. A strong positive correlation was found between the number of years an antimicrobial had been used on the farms (bacitracin had the longest reported use time of nine years, while amoxicillin-clavulanic acid, marbofloxacin, and gentamicin were reported to be used during only one year) and its corresponding resistance rate (Pearson correlation; r = 0.88, *p* < 0.001). Resistance was also positively correlated with the number of antimicrobials used per farm (Pearson correlation; *p* = 0.02), but no correlation was observed with the farm’s biosecurity score (Pearson correlation; *p* = 0.578). Notably, resistance to amikacin—an antimicrobial not reported as used in these herds—was also detected, which may indicate environmental contamination or horizontal gene transfer.

**Figure 1:**
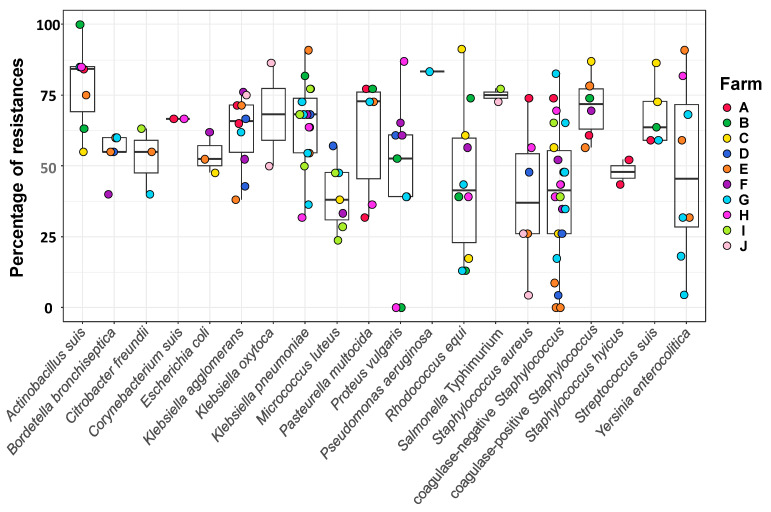
Percentage of antimicrobial resistance per bacterial species. Nasal isolates from sows from ten farms were tested for antimicrobial susceptibility. The results are presented as box-plots, with a horizontal line indicating the median value and the box representing the middle 50% of the observed values. The results are organized by bacterial species and represent the percentage of resistance (the number of tests giving a resistance result with respect to the total number of tests). The farms are represented with different colors following the legend on the right of the graph. Raw data can be found in Tables S1 and S3.

**Figure 2:**
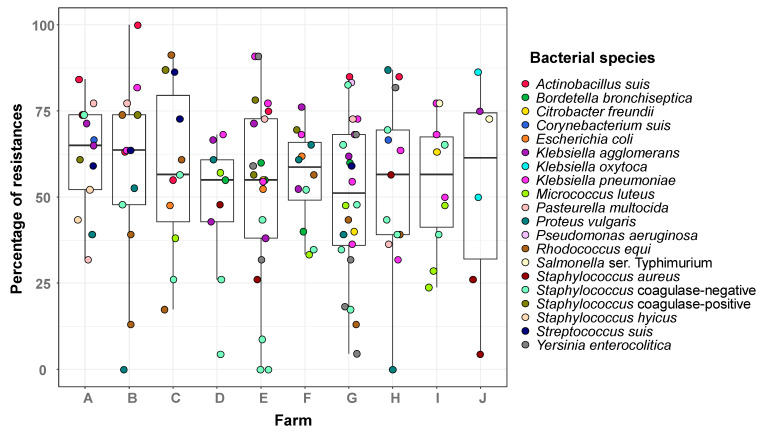
Percentage of antimicrobial resistance per farm. Nasal isolates from sows from ten farms were tested for antimicrobial susceptibility. The results are presented as box-plots, with a horizontal line indicating the median value and the box representing the middle 50% of the observed values. The results are organized by farm and presented in percentage of resistance (the number of tests giving a resistance result with respect to the total number of tests in each bacterial species). The bacterial species are represented with different colors following the legend on the right of the graph. Raw data can be found in Tables S1 and S3.

The phenotypic resistance profiles varied substantially among species (39–83%) and farms (47–65%) (Figure 1 and Figure 2; Supplementary Table S3). For example, coagulase-negative *Staphylococcus* isolates displayed a broad range of resistance, with isolates showing resistance to more than 80% of the tested AMBs and others showing susceptibility to all, while a narrower range of 59–86% of resistances was observed in *S. suis* isolates (Figure 1). The overall differences in the frequency of resistances among bacterial species were statistically significant (Kruskal–Wallis, *p* = 0.0015), mainly driven by differences between *A. suis* (isolates with resistances to 55–100% of the AM) and *M. luteus* (resistances to 24–57% of the AM) (z = 3.5, *p* = 0.037) and between *A. suis* and coagulase-negative *Staphylococcus* spp. (z = 3.8, *p* = 0.014). Although high resistance levels were also observed in *Salmonella* Typhimurium and *P. aeruginosa*, both were isolated from a limited number of farms, which may have limited statistical power to detect broader differences.

Additionally, resistance to ceftiofur, a third-generation cephalosporin of critical importance in both human and veterinary medicine, was observed in multiple taxa, including *Actinobacillus suis*, *S. aureus*, and *S. suis*, further underscoring potential public health risks. Finally, several significant associations between specific pathogens and on-farm antimicrobial practices were identified. For example, the isolation of *P. vulgaris* was significantly associated with penicillin administration (Chi-square, *p* = 0.0317), and the use of autogenous *S. suis* vaccine was associated with the isolation of *Rhodococcus equi* (Chi-square, *p* = 0.0410).

Several important opportunistic and pathogenic bacteria were isolated from sows without clinical signs of respiratory disease, including *P. multocida*, *B. bronchiseptica*, *Klebsiella pneumoniae*, *Pseudomonas aeruginosa*, *Staphylococcus aureus*, *Yersinia enterocolitica*, *Salmonella* Typhimurium, and *S. suis*. As expected, *A. pleuropneumoniae*—a primary swine respiratory pathogen—was isolated only from a sow presenting fever on farm J. These findings support the hypothesis that even apparently healthy animals can harbor significant antimicrobial-resistant pathogens, which represent a One Health concern.

Isolates with varying degrees of resistance were found in the different farms, but no differences were detected in the overall resistance levels among the farms (Krustal–Wallis, *p* = 0.94; Figure 2).

**Figure 3:**
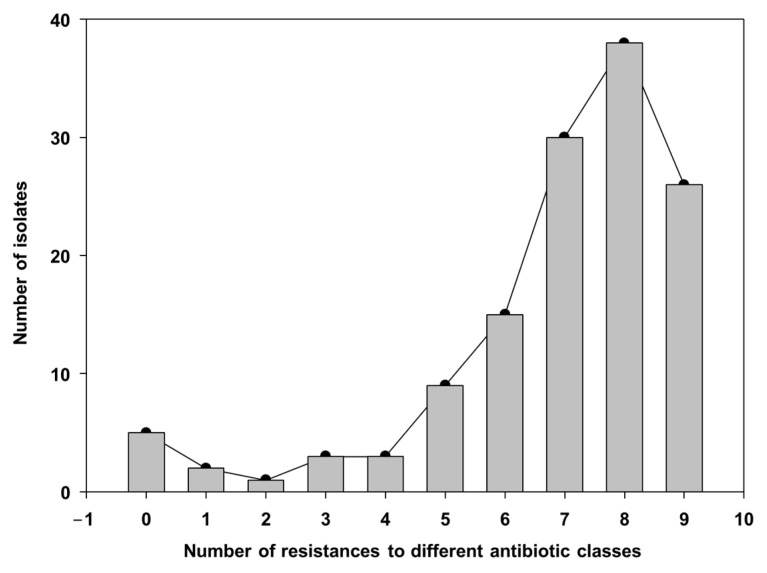
Distribution of number of resistances, including MDR (multi-drug resistance), in the bacterial isolates from the nasal cavities of sows obtained in this study.

When the resistance to multiple AMB families was examined, the majority of isolates showed multidrug resistance to 7–9 AMB classes (Supplementary Table S1; Figure 3). On the other hand, only five isolates did not show any resistance (two *Proteus* and three *Staphylococcus*), two isolates showed resistance to just one AMB family, and one isolate to two families. Some of the more frequent MDR profiles included resistance to bacitracin and penicillin combined with tetracycline (60% of isolates), doxycycline (57%), florfenicol (56%) or tylosin (55%).

## 4. Discussion

This cross-sectional study evaluates, for the first time, the antimicrobial resistance (AMR) of bacterial isolates collected from the nasal cavities of sows in the Federal District, Brazil. We also screened for the most common respiratory pathogens in swine production [24,25,26,27,28,29]. However, a high level of AMR was observed not only in pathogens but also in commensals. Globally, the rate of resistance to an AMB correlated with the number of years using the drug, and global resistance in a farm correlated with the number of AMBs used. In agreement with a previous report showing that biosecurity did not correlate with AMU [10], in this study, AMR was not associated with the biosecurity score.

Medicated feed is still a common strategy in many countries to control the occurrence of respiratory pathogens, such as *Mycoplasma*, *Pasteurella*, *Glaesserella* [9] and *S. suis* [26,27], although colonization by these pathogens does not always lead to disease. In line with a recent report [30] and our results, there are significant antimicrobial resistances among commensals. Herein, we found a multidrug resistance pattern in several commensals, such as *P. agglomerans* and coagulase-negative *S. aureus*. Also, Brazil has already characterized MDR coagulase-negative *Staphylococcus* involved in subclinical mastitis [31]. In China, a meta-analysis was conducted to investigate the epidemiology and antimicrobial resistance rates of coagulase-negative *Staphylococcus*, associated with bovine mastitis, and found that the majority of the isolates were resistant to beta-lactams [32].

All farms in this study used metaphylactic amoxicillin for sows, an AMB commonly used to treat *S. suis* infections, a zoonotic pathogen widely distributed in pig farms [33,34]. Pigs are usually colonized by more than one serotype of *S. suis*, but only a few strains can produce disease [27]. Beta-lactam-resistant *S. suis* strains are primarily found in commensal sites [33], while the majority of clinical *S. suis* isolates remain sensitive to amoxicillin [27]. Similarly, most of the *S. suis* isolated from the nasal swabs in this study were sensitive to amoxicillin, probably due to the lack of widespread production of beta-lactamases by this bacterium [26]. On the other hand, *S. suis* in this study presented AMR to other classes of AMBs, including quinolone, cephalosporin, and tetracycline, in agreement with recent reports that found high levels of resistance in *S. suis* strains isolated from clinically healthy sows in China (91.7% for tetracycline, 86.7% for sulfamethoxazole, 67.2% for erythromycin and 59.1% for trimethoprim/sulfamethoxazole) [26]. Also, *S. suis* isolates from Australia showed high resistance frequencies for tetracycline (99.3%) and erythromycin (83.8%) [24]. In addition, all nasal *S. suis* isolates in our study showed resistance to florfenicol and clindamycin, which is a higher frequency than previously reported [26,27]. It is worth noting that we found *S. suis*, *A. suis*, and *S. aureus* isolates that were resistant to ceftiofur, a third-generation cephalosporin.

These findings are concerning, since third- and fourth-generation cephalosporins are considered critically important AMBs in human medicine [35]. In addition, resistance to ceftiofur has been described in *P. multocida* of cattle origin [35], and *P. multocida* isolates of wildlife origin [36], reinforcing the fact that AMR is a multifactorial problem, with intrinsic links in the human, animal, and environmental interface. Furthermore, *Pasteurellaceae* isolates from wild and domestic animals in an alpine ecosystem in northeastern Spain exhibited similar levels of resistance to macrolides [36]. Here, we also found a high level of frequency of resistance to macrolides, particularly tylosin. As a cautionary note, tylosin was used in the medicated feed for sows on Farm I, and all isolates from this farm showed resistance to this antimicrobial.

In this context, the new regulation implemented in 2024 (Decree No. 12.031/2024), [37] introduced an updated regulatory framework in Brazil for feed production, including medicated feeds, with the aim of preventing misuse, safeguarding animal health, and mitigating antimicrobial resistance. Currently, authorization and licensing are key requirements for the manufacture of medicated feed and its use requires a veterinarian’s prescription. Ongoing government initiatives are aimed at educating veterinarians and producers on the responsible use of medicated feed, reflecting the latest legislative changes [37].

An alternative to AMU to control the spread of pathogens and infectious diseases is the use of vaccines. In this study, the use of autogenous vaccines against *S. suis* was positively associated with *Rhodococcus equi* isolation. While the statistical result between *S. suis* vaccine and *R. equi* isolation is intriguing, future studies about microbiota shifts may help to better understand the underlying mechanisms. We could hypothesize that the vaccine affected the composition of the microbiota, probably reducing *S. suis* and potentially shifting the balance of *R. equi* colonization. On the other hand, the effect of the vaccine may be indirect, through the immune system. This protective effect of vaccines was observed with the autogenous *P. multocida* vaccines, which reduced the incidence of lung lesions reported by the slaughterhouse, highlighting the promising role of targeted bacterial vaccination in managing respiratory disease and reducing AMU.

Antimicrobial misuse is concerning not only because it contributes to the emergence of resistance, but also for the deleterious impact of these drugs on microbiota communities. The nasal microbiota contributes to respiratory health [13], mainly through pathogen exclusion and immune system stimulation [38,39], provided by a diverse microbial community [40]. Mou et al. [41] found that oral oxytetracycline (which was used in Farm A) had a greater effect on the diversity and disruption of the microbiota than the intramuscular route. They described different dosing regimens of oxytetracycline associated with shifts in the nasal microbiota [41]. Antimicrobial treatments in sows could be a significant cause of dysbiosis in the offspring. Bonillo-Lopez et al. [42] showed that sow treatment reduces the nasal bacterial load of sows and alters the composition of the nasal microbiota of piglets, showing unusual taxa in their nasal microbiota. In this study, we found a potential link between penicillin supplementation and the presence of *P. vulgaris* in the nasal cavity. AMBs can alter microbiota, reducing competition and allowing the proliferation of opportunistic bacteria [38]. Furthermore, *Proteus* is known for its ability to acquire resistance genes through plasmids, transposons, and other mobile genetic elements, facilitating their survival in environments with AMB pressure [5,6,7,8]. If other susceptible bacteria are eliminated, pathobionts can proliferate [38]. Since amoxicillin and penicillin, being Beta-lactams, target preferentially Gram-positive bacteria, Gram-negative bacteria may gain a competitive advantage in the microbiome environment [12]. Still, some farm managers in this survey alternated penicillin, florfenicol, tylosin, clindamycin, tetracycline, enrofloxacin, and oxytetracycline with amoxicillin in the feed, disregarding that metaphylactic treatments can be avoided without negatively impacting production [39].

Although the interactions between bacterial species within the swine nasal microbiota are not yet fully understood, some commensal members may play a protective role by competing with respiratory pathogens [40]. Notably, changes in the nasal microbiota have been observed following different oxytetracycline dosing regimens in pigs [41]. Furthermore, intensive antibiotic treatment of sows with parenteral crystalline ceftiofur and tulathromycin has been shown to alter the composition of the nasal microbiota in their offspring [42]. On this context, Mahmmod et al. [43] estimated a statistically significant association for *G. parasuis* colonization, where *Bacteroidaceae* and *Mycoplasmataceae* in the nasal microbiota of piglets were likely to prevent colonization by virulent *G. parasuis*, whereas *Chitinophagaceae* and *Streptococcaceae* were associated with a higher likelihood of colonization by virulent *G. parasuis*. Similarly, pig carriers or non-carriers of *S. aureus* presented a distinct nasal microbiome and probably differential network involving complex interactions [29].

Given its importance, commensal microbiota should not act as a reservoir of resistance genes [44]. Testing commensal communities can be a valuable tool for AMR surveillance, as these organisms may serve as early indicators of antimicrobial resistance trends in animal populations. In our study, we observed relevant resistance patterns in key bacterial species commonly found in both commensal and pathogenic microorganisms, such as *Staphylococcus* spp., *E. coli*, and *Klebsiella* spp. These pathogens are of particular concern within the One Health framework due to their capacity to acquire and disseminate resistance genes across human, animal, and environmental interfaces.

A limitation of this study is that resistance was assessed only phenotypically. Incorporating genotypic methods, such as PCR detection of resistance genes or whole-genome sequencing, would provide deeper insights into the resistance mechanisms and potential for horizontal gene transfer. While bacterial isolation and antimicrobial susceptibility testing are time-consuming and may not be suitable for use in current farm practices [17], multiplex PCR has the potential to be a faster technique implemented for a national antimicrobial resistance surveillance program [45]. In any case, susceptibility tests [18,19,20,21,22,23,24,25,26,27,28,29,30,31,32,33,34,35,36,37,38,39,40,41,42,43,44,45,46] will help the farm manager choose the appropriate AMB in the event of disease outbreaks [47].

## 5. Conclusions

In this study both the duration and diversity of antimicrobial use in feed were associated with increased resistance rates among bacterial colonizers in the nasal microbiota of sows. Phenotypic resistance was observed for all antimicrobials tested, including the detection of multidrug-resistant isolates. These findings highlight the potential of nasal swab sampling as a complementary tool for antimicrobial resistance surveillance in swine herds. Additionally, the implementation of vaccination protocols—particularly the use of autogenous vaccines—was associated with a reduced incidence of respiratory lesions, reinforcing their strategic value in disease prevention and antimicrobial stewardship.

## Figures and Tables

**Table 1:** Farm biosecurity evaluation by scoring preventive measures.

Farm	Farm Isolation	Swine Herds Distance	Road Distance	Breeders Reposition	Quaran-tine	Vectors Control	Type of Feed	Feed Transport	Vehicle Disinfection	Human Access	Bio Score *
A	1	0	0.5	1	0.5	1	1	1	1	1	**8**
B	0.75	1	0.75	1	0	0.25	1	1	0.25	1	**7**
C	1	0.75	0.25	1	1	1	1	1	1	1	**9**
D	0.5	1	0.75	0.5	0	0.5	1	1	0.75	1	**7**
E	1	1	1	0	0	1	1	1	0	0	**6**
F	0.5	1	0.75	1	0	0.5	1	1	0.25	1	**7**
G	1	1	1	0	0	1	1	1	0.5	0.5	**7**
H	0.25	1	0.75	1	0	1	1	1	0.5	0.5	**7**
I	1	1	0.25	1	0	1	1	1	0.25	0.5	**7**
J	1	0	1	0	0	1	1	1	0.5	0.5	**6**

* Biosecurity score is composed of biosecurity modules (columns). Scale (0, 0.25, 0.5, 1): 0 = None, 0.25 = Low, 0.5 = Moderate, 1 = High. Bioscore (0–10) is the sum of scores across categories, with higher values for greater biosecurity.

**Table 2:** Antimicrobials used for susceptibility testing, disk diffusion concentration, and CLSI interpretive criteria for resistance pattern.

Pharmacologic Class	Antimicrobials	Concentration in Disk
Aminoglycoside	Amikacin (AMI)	30 µg
	Gentamicin (GEN)	10 µg
	Neomycin (NEO)	30 µg
Amphenicol	Florfenicol (FLF)	30 µg
Beta-lactam	Amoxicillin + Clavulanic acid (AMC)	20 µg
	Amoxicillin (AMO)	30 µg
	Ampicillin (AMP)	10 µg
Penicillin	Penicillin (PEN)	30 µg
Cephalosporine	Cephalothin (CFL)	30 µg
	Cephalexin (CFE)	30 µg
	Ceftiofur (CFT)	30 µg
Quinolones	Enrofloxacin (ENO)	5 µg
	Marbofloxacin (MBO)	5 µg
	Norfloxacin (NOR)	10 µg
Lincosamide	Clindamycin (CLI)	2 µg
Macrolide	Erythromycin (ERI)	15 µg
	Tylosin (TLS)	60 µg
	Tulathromycin (TUL)	30 µg
Tetracycline	Tetracycline (TET)	30 µg
	Doxycycline (DOX)	30 µg
Polypeptide	Bacitracin (BC)	10 µg
Sulphonamide	sulfametoxazol (SUL)	300 µg
	sulfametoxazol-trimetoprim (SUT)	25 µg

**Table 3:** Farm, antimicrobial agents in metaphylactic treatment, vaccine protocols and production type.

Farm	AMB Agents	Vaccines	Production Type
A	AMO, CLI, TET, ENO, OXY	*M. hyopneumoniae*, *circovirus*, *P. multocida*, *S.* ser. Typhimurium	One-site-herd: piglet unit production
B	AMO, FLF, PEN	*M. hyopneumoniae*, *circovirus*, *G. parasuis*, *S. suis*.	One-site-herd: piglet unit production
C	AMO, FLF	*P. multocida*, *B. bronchiseptica*, *G. parasuis*, *S.* ser. Typhimurium, *E. coli.*	Two-site-herd: piglet and gilt and young boar production
D	AMO, FLF	*G. parasuis*	One-site-herd: piglet unit production
E	AMO	None	Farrow-to-finish: piglet to hog-finished production
F	AMO, FLF, PEN	*P. multocida*, *S.* ser. Typhimurium, *S. suis*.	One-site-herd: piglet unit production
G	AMO	*None*	Farrow-to-finish: piglet to hog-finished production
H	AMO, FLF	*M. hyopneumoniae*, *circovirus*, *P. multocida*, *S.* ser. Typhimurium, *S. suis*	One-site-herd: piglet unit production
I	AMO, FLF, TYL	*M. hyopneumoniae*, *circovirus*, *P. multocida*, *G. parasuis*, *S.* ser. Typhimurium	One-site-herd: piglet unit production
J	AMO, CLI	*P. multocida*, *G. parasuis*	Farrow-to-finish: piglet to hog-finished production

AMO: amoxicillin, CLI: clindamycin, TET: tetracycline, ENO: enrofloxacin, OXY: oxytetracycline, PEN: penicillin, FLF: florfenicol, TYL: tylosin.

**Table 4:** Pathogen detection by PCR or bacterial culture in nasal samples from sows.

Farm	PCR	Culture
A	*P. multocida*	*A. suis*; *P. multocida*; *S. suis*.
B	*P. multocida*; *G. parasuis*	*A. suis*; *P. multocida*; *S. suis*.
C	*G. parasuis*	*A. suis*; *S. suis*
D	*P. multocida*	*B. bronchiseptica*
E	*P. multocida*	*A. suis*; *B. bronchiseptica*; *P. multocida*; *Yersinia enterocolitica*
F	*G. parasuis*	*B. bronchiseptica*
G	*P. multocida*	*A. suis*; *B. bronchiseptica*; *P. multocida*; *S. suis*; *Y. enterocolitica*
H	*P. multocida*	*A. suis*; *P. multocida*; *Y. enterocolitica*
I	*G. parasuis*	*A. suis*; *S. suis*
J	*A. pleuropneumoniae*	*S.* ser. Typhimurium; *S. aureus*

## Data Availability

The original contributions presented in this study are included in the article/Supplementary Materials. Further inquiries can be directed to the virginia.aragon@irta.cat and luciana.rigueira@gmail.com.

## Supplementary Materials

The following supporting information can be downloaded at: https://www.mdpi.com/article/10.3390/microorganisms13061354/s1, Table S1: Resistances found in each isolate are indicated with a 1 below the antimicrobial agent tested (atm) 0 indicated susceptibility; Table S2: Antimicrobial agent (atm); Period in sow´s farm (years); Results of resistance (R), n° (%); Table S3: Number of isolates of each bacterial species obtained in each farm and the associate percentage of resistance, calculated as the number of tests giving a resistance result with respect to the total number of tests in each bacterial species (resistance per species) or each farm (resistance per farm).
